# Texture analysis on MR images helps predicting non-response to NAC in breast cancer

**DOI:** 10.1186/s12885-015-1563-8

**Published:** 2015-08-05

**Authors:** N. Michoux, S. Van den Broeck, L. Lacoste, L. Fellah, C. Galant, M. Berlière, I. Leconte

**Affiliations:** 1 Radiology Department, IREC (Institute of Experimental and Clinical Research) - IMAG, Université Catholique de Louvain, Cliniques Universitaires Saint-Luc, Avenue Hippocrate 10, Brussels, B1200 Belgium; 2 Radiology Department, Cliniques Universitaires Saint-Luc, Avenue Hippocrate 10, Brussels, B1200 Belgium; 3 Surgery Department, Cliniques Universitaires Saint-Luc, Avenue Hippocrate 10, Brussels, B1200 Belgium; 4 Pathology Department, Cliniques Universitaires Saint-Luc, Avenue Hippocrate 10, Brussels, B1200 Belgium

**Keywords:** Breast cancer, Neoadjuvant chemotherapy, MRI, Texture analysis

## Abstract

**Background:**

To assess the performance of a predictive model of non-response to neoadjuvant chemotherapy (NAC) in patients with breast cancer based on texture, kinetic, and BI-RADS parameters measured from dynamic MRI.

**Methods:**

Sixty-nine patients with invasive ductal carcinoma of the breast who underwent pre-treatment MRI were studied. Morphological parameters and biological markers were measured. Pathological complete response was defined as the absence of invasive and in situ cancer in breast and nodes. Pathological non-responders, partial and complete responders were identified. Dynamic imaging was performed at 1.5 T with a 3D axial T1W GRE fat-suppressed sequence. Visual texture, kinetic and BI-RADS parameters were measured in each lesion. ROC analysis and leave-one-out cross-validation were used to assess the performance of individual parameters, then the performance of multi-parametric models in predicting non-response to NAC.

**Results:**

A model based on four pre-NAC parameters (inverse difference moment, GLN, LRHGE, wash-in) and *k*-means clustering as statistical classifier identified non-responders with 84 % sensitivity. BI-RADS mass/non-mass enhancement, biological markers and histological grade did not contribute significantly to the prediction.

**Conclusion:**

Pre-NAC texture and kinetic parameters help predicting non-benefit to NAC. Further testing including larger groups of patients with different tumor subtypes is needed to improve the generalization properties and validate the performance of the predictive model.

## Background

Neoadjuvant chemotherapy (NAC) has a major role in the treatment of breast cancer [1, 2]. Several trials comparing adjuvant chemotherapy and NAC demonstrated that long-term relapse-free and overall survival outcomes were the same [3]. However, NAC has advantages compared with adjuvant chemotherapy. NAC can safely downstage tumor so that conservative surgery can be performed instead of mastectomy [4, 5]. Importantly, NAC is the only way to study the effect of systemic chemotherapy in vivo and to identify prognostic factors. However, the rate of response to NAC is limited and dependent on the subtypes of cancer [6–12]. It has been recently reported that pathological complete response (pCR) obtained after NAC is a suitable surrogate endpoint for disease-free survival in patients with luminal B/Human Epidermal growth factor Receptor 2 (HER2) -negative, HER2-positive (non-luminal) and triple negative tumors but not for those with luminal B/HER2-positive or luminal A tumors. However, the rate of pCR in these different breast cancer subtypes varies from 6 to 33 % [13]. Therefore, the identification of non-responding patients is important, especially as it may allow considering alternative therapeutic options.

The predictive value of Magnetic Resonance Imaging (MRI) and in particular of diffusion-weighted MRI [14–16], MR spectroscopy [17–19] or Dynamic Contrast-Enhanced MRI (DCE-MRI) [20–23] has been investigated. However, most of these studies were performed after the first courses of NAC. Some studies reported that certain pre-NAC semi-quantitative DCE parameters were significantly different in chemosensitive and chemoresistant breast lesions and may contribute to the prediction of disease-free survival and overall survival [24–26].

Alternative quantitative approaches such as visual texture analysis have been considered [27, 28]. Texture analysis allows for the description of the MR appearance of the tissues and of their changes in terms of fineness, coarseness, smoothness, granularity, homogeneity or periodicity [29]. These attributes are related to the local spatial distribution of the grey levels in the image matrix and can be captured by using metrics, called texture parameters. In texture analysis of MR images, it is assumed that the distribution of the grey levels results from the underlying ultrastructural properties of tissues affected by the disease processes-an assumption that has been validated by finding correlation between MRI texture patterns and tissue changes on histological analysis [30]. Numerically, texture can be described by using n^th^-order statistics, spatial frequency or structural primitives, the first two approaches being the most commonly used. A practical description of the concepts and methodologies for texture analysis of MR images has been proposed by Hajek et al. [31]. First studies in breast MRI, while remaining to be validated, showed that certain pre-treatment texture parameters (based on high order statistics) may help evaluate breast tumor response to NAC [32–34].

The aim of the study is to assess the value of pre-NAC imaging parameters to predict non-responders to NAC. To this purpose, texture, kinetic and BI-RADS (Breast Imaging-Reporting and Data System) parameters were studied from baseline MRI. Thence, a three-step assessment was undertaken. First, texture parameters were compared in healthy breast tissues and in tumor lesions. Secondly, the performance of individual parameters in predicting pathological non-response to NAC was assessed. Thirdly, parameters were combined into multi-parametric models. The predictive performance of these multi-parametric models was then assessed after cross-validation.

## Methods

### Patients

This two-years retrospective study was approved by our institutional ethical committee (Comité d’Ethique hospitalo-facultaire, Cliniques Universitaires Saint-Luc, http://www.comite-ethique-ucl-saintluc.be/). Written informed consent from the patients was not required. All patients had an invasive breast carcinoma diagnosed on core-biopsy specimen. To obtain a homogeneous histological sample for texture analysis, only invasive ductal carcinomas (IDC) with and without ductal carcinoma in situ (DCIS) were included in this pilot study. The mean number of cancers-newly diagnosed in our institution was 296 per year. Seventeen percent of patients with invasive cancers received NAC. The percentage of in situ (DCIS and LCIS) was comprised between 17 to 21 %.

A baseline MRI as well as a pre-operative MRI to evaluate response to NAC was performed in all patients. After multidisciplinary breast cancer tumor board decision, all patients underwent NAC, consisting of 4 cycles of cyclophosphamide/anthracyclines followed by 4 cycles of taxanes [2, 3] and trastuzumab in case of HER2+ tumor. Cycles were administrated every 3 weeks. All patients had surgery three to four weeks after completing NAC. As a result, the delay between diagnosis and surgery was approximately 6 months.

Patients with incomplete pathological and radiological data (*n* = 6) and severe artifacts on MRI images (e.g. respiratory motion and body movement) (*n* = 3) were excluded. Overall, this retrospective study included 69 patients with IDC (median age 54 years, range 22–72 years). Estrogen receptor (ER), progesterone receptor (PgR) and, HER2 status as well as the mitotic factor Ki67 were available on percutaneous biopsies. Patients’ characteristics are listed in Table 1.

**Table 1:** Patients characteristics (*n* = 69). Number and proportions within the whole population are given

Characteristics	Values
Median age (range)	54 (22–72)
**BI-RADS feature**	
Mass	39 (57 %)
non mass	30 (43 %)
**Histological grade**	
IDC 1	0
IDC 2	25 (36 %)
IDC 3	44 (64 %)
**Subtypes**	
Luminal A	13 (19 %)
Luminal B/HER2-	25 (36 %)
Luminal B/HER2+	15 (22 %)
Non luminal/HER2+	10 (14 %)
Triple-negative	6 (9 %)
**Receptor status**	
ER positivity	52 (75 %)
PgR positivity	42 (61 %)
Ki67 > 14 %	52 (75 %)
HER2 positivity	26 (38 %)
Triple-negative cancer rate	6 (9 %)

*IDC* invasive ductal carcinoma, *ER* estrogen receptor, *PgR* progesterone receptor, *HER2* epidermal growth factor receptor 2

### Pathological and biological analysis

Breast tissues sampled for histopathological analysis were sectioned at the macroscopic level transversally in order to produce 10 mm slices. A dedicated breast pathologist analyzed each lesion at the microscopic level, describing first the size of every residual cancerous foci and then classifying these into three categories according to the NSABP 18 criteria [35]: pathological complete (CR), partial (PR) and non-response (NR). In case of a single mass lesion with a concentric response, the size of the residual tumor was measured. In case of a single masse lesion with a fragmented response, i) the overall dimension of the foci is given when foci are adjacent, ii) each foci is measured when foci are distant and the overall sum is given. In case of a non-mass lesion with fragmented response, the overall size is given.

The density of tumor cells, compared to the previous biopsy, was also analyzed, allowing the classification of the tumor following the grading system of Miller-Payne (5 grades). The tumor grade was evaluated with the Nottingham score.

A pathological complete response was defined as the absence of invasive and in situ cancer in breast and nodes. A partial response was defined as a decrease of invasive cancer exceeding 30 %. A non-response was defined as a decrease of invasive cancer lower than 30 %. At histological analysis, 14 patients were thus classified as CR, 36 as PR and 19 as NR.

All biological markers were evaluated on percutaneous biopsies. As regards immunohistochemical assessments, IDCs were classified according to their receptor status. ER and PgR were considered as negative when <10 % nuclei stained positive [36]. For all lesions, the results for HER 2 expression by immunohistochemical analysis were scored as 0, 1+, 2+ and 3+. Only 3+ specimens were immediately considered as HER2-positive. A hybridization technique was performed when analysis score was 2+. Both negative and 1+ were considered as negative. The mitotic activity marker Ki67 was considered as positive when expressed by more than 14 % of tumor cells [13]. Correlation between sensitivity of breast cancer to NAC and receptor status is given in Table 2.

**Table 2:** Association between pathologic responsiveness of breast cancer to NAC and receptor status

Pathologic response	NR	CR	PR	PR + CR	*p*-value^a^
**BI-RADS**					
Mass	12 (31 %)			27 (69 %)	0.51
non Mass	7 (23 %)			23 (77 %)	0.51
**Biological markers**					
ER positivity	15 (29 %)	9 (17 %)	28 (54 %)	37 (71 %)	0.70
PgR positivity	14 (33 %)	4 (10 %)	24 (57 %)	28 (67 %)	0.19
Ki67 > 14 %	11 (21 %)	11 (21 %)	30 (58 %)	41 (79 %)	0.05
HER2 positivity	4 (15 %)	8 (31 %)	14 (54 %)	22 (85 %)	0.09
**Subtypes**					
Luminal A	8 (62 %)	0	5 (38 %)	5 (38 %)	0.005
Luminal B/ HER2 –	4 (16 %)	5 (20 %)	16 (64 %)	21 (84 %)	0.11
Luminal B/HER2 +	3 (20 %)	4 (27 %)	8 (53 %)	12 (80 %)	0.49
Non-luminal/HER2 +	1 (10 %)	4 (40 %)	5 (50 %)	9 (90 %)	0.20
Triple-negative cancer rate	3 (50 %)	1 (17 %)	2 (33 %)	3 (50 %)	0.25
**Histological grade**					
IDC 2	5 (20 %)	3 (12 %)	17 (68 %)	20 (80 %)	0.31
IDC 3	14 (32 %)	11 (25 %)	19 (43 %)	30 (68 %)	0.31

The number and proportions of NR, CR, PR and PR + CR patients with a given feature within all patients having this feature are given. The statistical significance of the relationship between response (NR or PR + CR) and features is then assessed (*p*-value^a^). If a *p*-value < 0.05 is observed for a given feature, then we can conclude that patients’ response is associated to that feature. If a *p*-value > 0.05 is observed, then the null hypothesis that there is no association, cannot be rejected. Subtype Luminal A is the only feature showing a significant association with response

*BI-RADS* breast imaging-reporting and data system, *NR* non response, *CR* complete response, *PR* partial response, *ER* estrogen receptor, *PgR* progesterone receptor, *Ki67* cellular marker for proliferation based on monoclonal antibody Ki-67, *HER2* human epidermal growth factor receptor 2, *HR* hormone receptor, *IDC* invasive ductal carcinoma

^a^Significance of the association between response (NR or PR + CR) and features (Fisher’s exact test)

### MRI sequence

MRI examinations were performed using a 1.5 T whole body imaging system (Gyroscan Intera, Philips Medical System, The Netherlands) and a breast coil. Patients were imaged in the prone position with T2-weighted and diffusion-weighted imaging (DWI) (b0, b600) sequences, and a 3D gradient echo axial T1-weighted sequence with fat suppression (SPAIR). Scan parameters were TR/TE = 4.8/2.4 ms, flip angle = 10°, FOV = 355 × 355 mm, matrix 320 × 320, slice thickness 2.5 mm, voxel size 0.65 × 0.65 × 1.25 mm after reconstruction. The anatomic study was followed by a dynamic study. Patients received 0.1 mmol/kg of gadobenate dimeglumine (Multihance, Bracco Imaging, Germany) followed by 30 mL saline flush injected at a rate of 2 mL/s with an automated injector. One pre- and five post-injection images were acquired with a temporal resolution of approximately 60 s. The total acquisition time for the protocol was about 6 min. Analyses were performed on subtracted images, i.e. the residual difference image obtained after the second post-contrast image has been subtracted from the pre-contrast image.

### Image analysis

**Fig. 1:**
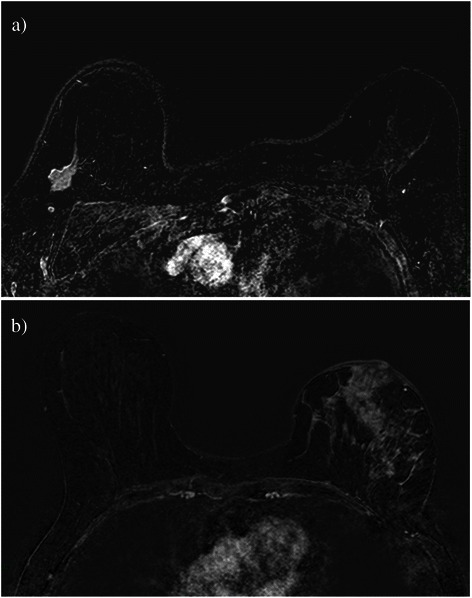
Axial subtracted images. According to the BI-RADS MR lexicon, the tumor is described as, **a** ovalar mass with spiculated margins and a homogenous enhancement in the upper external quadrant, or **b** retro-areolar non mass lesion, showing a cobblestone-like pattern with nipple invasion and skin thickening

Magnetic resonance images in 69 patients were reviewed consensually by a trainee and two experienced radiologists (10 and 15 years of breast MRI experience respectively) without knowledge of the pathological findings or mammographic and sonographic data, by using the American College of Radiology BI-RADS MR lexicon [37]. Lesions were categorized into mass enhancement and non-mass enhancement (Fig. 1 and Table 2). The uni- or multifocal character of the lesion was reported. In case of multifocal lesion, only the findings of the largest lesion were recorded. The slice exhibiting the largest dimension of the lesion on the second post-contrast image (enhancement peak) was chosen for analysis. This criterion was applied in case of mass enhancement or non-mass enhancement.

**Fig. 2:**
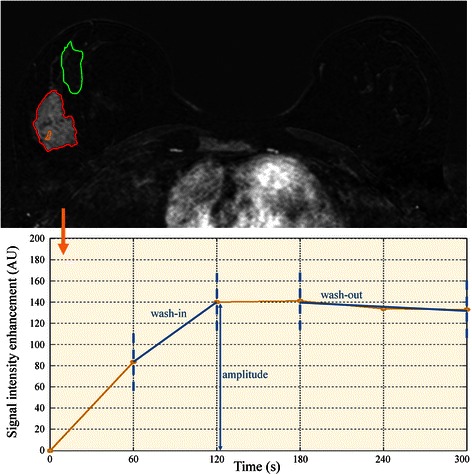
Top, axial fat-suppressed T1 weighted imaging (time corresponding to the second post-contrast image). Two large ROIs, one encompassing the lesion (*in red*) and one encompassing normal breast tissues (*in green*), were defined for visual texture analysis. A small ROI (*in yellow*) in the brightest part of the lesion was also defined to study the kinetics of the contrast agent. Bottom, the signal intensity *vs* time curve (temporal sampling 60 s) corresponding to the small ROI (from which kinetic parameters are derived) is displayed. Amplitude was calculated from the maximum enhancement peak, the wash-in parameter from the up-slope measurement (between the maximum enhancement peak and the preceding time point) and the wash-out parameter from linear regression performed on the last three time points of the signal intensity versus time curve

For kinetic analysis, a small region of interest (ROI) corresponding to the most enhancing area of the lesion was drawn (Fig. 2). The size of the ROI always included more than nine pixels [38]. According to the BI-RADS guidelines, characteristics of the signal intensity *vs* time curve (i.e. the maximal amplitude, the wash-in and the delayed phase pattern *via* the wash-out parameter) were assessed.

**Fig. 3:**
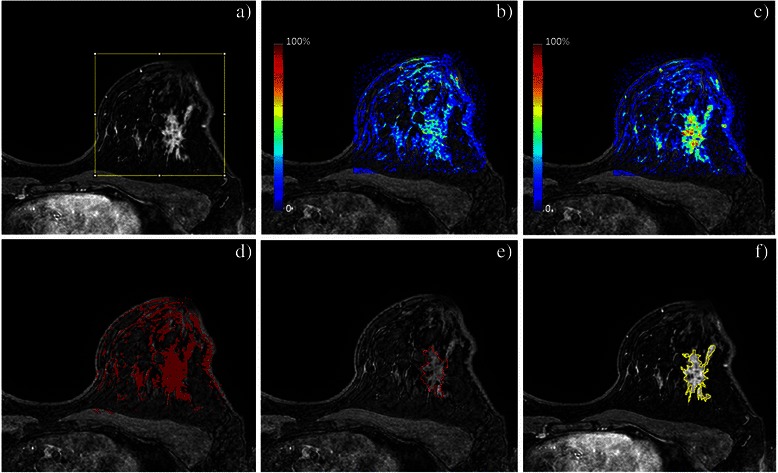
Automated segmentation of the tumor lesion. A rectangular area covering the breast is placed (**a**). Pixel-level calculation of parameters wash-in (**b**) and amplitude (**c**) is performed. Pixels are partitioned into *k* = 2 clusters (**d**). Morphological opening is applied to preserve the largest region of contiguous pixels with the same behavior in amplitude and wash-in only (**e**). Comparison with the manual delineation of the lesion shows an overall good agreement (**f**)

For texture analysis, a first ROI delimiting healthy tissues was drawn. Healthy tissues were delimited in a remote area of the lesion in the same breast, or in the contralateral breast in case of very large lesions. Based on texture differences observed between fat and healthy tissues (data not shown), healthy tissues were defined as fibroglandular tissues excluding fatty tissues. This distinction was always feasible as none of the patients studied had exclusively fat breast. A second ROI delimiting the lesion was drawn. The lesion was defined as the largest area with a high enhancement, excluding macro vessels. As this definition may be operator dependent, an automated segmentation was also implemented (Fig. 3). In brief, a rectangular ROI was defined in order to cover the whole breast. For each pixel within this ROI, parameters amplitude and wash-in were calculated. A *k*-means clustering algorithm was used to partition the pixels into 2 clusters (lesion and non-lesion) [39]. Then, a morphological opening was applied to remove isolated groups of pixels. The result of the segmentation was the largest region of contiguous pixels with the same behavior in amplitude and wash-in. This result was validated by comparison with the ROI drawn manually.

**Fig. 4:**
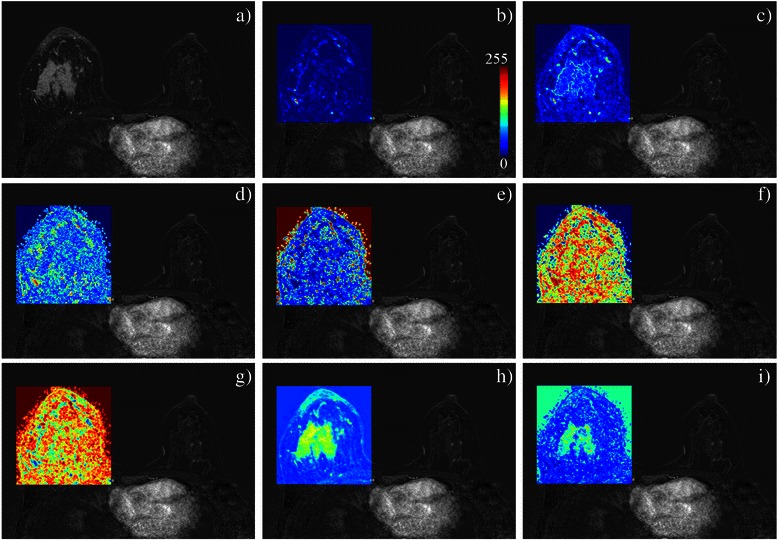
Pixel-level analysis of breast MRI texture in a CR patient with a mass enhancement. Are respectively displayed, **a** the axial subtracted image and the maps based on **b** contrast, **c** correlation, **d** difference variance, **e** energy, **f** entropy, **g** inverse differential moment (which is correlated with the homogeneity parameter), **h** sum average and **i** sum variance from the GLCM, with mean value estimated on a 3x3 neighbourhood around the pixel of interest then normalized on the 0–255 range. Individual texture parameters reveal different local and regional statistical properties of the grey level intensity between (and respectively within) breast lesions and normal parenchyma. Combination of all or parts of the texture parameters helps classifying patients according to their response to NAC

The visual texture of breast tissues was assessed from the grey level co-occurrence matrix (GLCM) and the run length matrix (RLM) [29, 40]. From the GLCM, nine textural features describing the grey levels interdependence in the image were estimated (Fig. 4). Computation parameters were: distance of one pixel between two neighbouring pixels, average of the angular relationships on the four main directions, five bits of grey levels. From the RLM, eleven textural features describing the distribution of runs of grey levels in the image were estimated with the same computation parameters. The mean value (over all pixels in the ROI studied) of the textural features was estimated. The list of studied parameters is given in Table 3.

**Table 3:** List of parameters used for breast lesion characterization

Parameter type		Parameter description
**Kinetic**		
1	Wash-in rate	Rate of contrast material uptake
2	Maximal amplitude	Maximal contrast enhancement
3	Wash-out rate	Rate of contrast enhancement washout
**Geometric (according to BI-RADS lexicon)**		
4	Mass	3D space-occupying lesion that comprises one process,
		usually round, oval, lobular or irregular in shape
5	non Mass	Enhancement of an area that is not a mass
**Texture**		
6^a^	Energy	Measure of local uniformity of grey levels
7^a^	Entropy	Measure of randomness of grey levels
8^a^	Contrast	Measure of the amount of grey levels variations
9^a^	Homogeneity	Measure of local homogeneity. It increases with less contrast
10^a^	Correlation	Measure of linear dependency of grey levels of neighbouring pixels
11^a^	Inverse difference moment	Measure of local homogeneity of the grey levels
12^a^	Sum average	Measure of overall image brightness
13^a^	Sum variance	Measure of how spread out the sum of the grey levels of voxel pair is
14^a^	Difference in variance	Measure of variation in the difference in gray levels between voxel pairs
15^b^	SRE	Short Run Emphasis (first property of run-length distribution)
16^b^	LRE	Long Run Emphasis
17^b^	GLN	Gray-Level Nonuniformity
18^b^	RLN	Run-Length Nonuniformity
19^b^	RP	Run percentage
20^b^	LGRE	Low Gray-Level Run Emphasis
21^b^	HGRE	High Gray-Level Run Emphasis
22^b^	SRLGE	Short Run Low Gray-Level Emphasis
23^b^	SRHGE	Short Run High Gray-Level Emphasis
24^b^	LRLGE	Long Run Low Gray-Level Emphasis
25^b^	LRHGE	Long Run High Gray-Level Emphasis

^a^Parameters derived from the co-occurrence matrix [29]

^b^Parameters derived from the run length matrix [40]

*3D* three-dimensional, *BI-RADS* breast imaging reports and data system

### Statistical analysis

Numerical variables are expressed as median and range (95 % CI on the median). The three-step comparative approach was conducted as follows. First, texture parameters were compared in healthy breast tissues *vs* tissues showing characteristics of a malignant lesion. A Wilcoxon rank-sum test was performed. This non-parametric test was chosen as the normality of the data distribution was not verified (on the basis of the D’Agostino-Pearson test).

Secondly, texture, kinetic, BI-RADS and biological parameters were compared in NR *vs* PR + CR individually. A mid-P approach of Fisher’s exact test was performed for assessing the relationship between response (NR or PR + CR) and features [41]. The performance of each parameter in predicting non-response to NAC was assessed by using receiver operating characteristic (ROC) curves and by comparing Area Under the ROC Curves (AUC) [42].

Thirdly, texture, kinetic, BI-RADS and biological parameters were combined. Two multi-parametric classifiers, each belonging to one of the two classes of algorithms in machine learning (supervised and unsupervised), were tested: a logistic regression model [43] and a *k*-means clustering algorithm based on a nearest-cluster approach [39]. The *k*-means algorithm was parameterized with a number of final clusters equal to 2, 2 random observations to choose the initial cluster centroid positions, 30 replicates and with the L1 distance to calculate the distance between centroid clusters. As one cannot know *a priori* how many and which parameters are important to the classification, all possible combinations of 2 to 26 parameters among 26 parameters (20 texture parameters, 3 kinetic parameters, the mass/non-mass enhancement, Ki67 > 14 %, HR/HER2) were submitted to the classifiers successively.

To estimate how accurately the predictive models would perform in practice, a leave-one-out cross validation was applied [44]. The cross validation works by leaving one observation (i.e. one patient data) out each time the classifier is trained. Thus, the observation can be used to test the classifier accuracy. The operation is then carried out for all observations. Hence, the percentage of NR patients classified correctly (i.e. the classifier sensitivity, Se) and the percentage of PR + CR patients classified correctly (i.e. the classifier specificity, Sp) were estimated. Se and Sp were finally used to identify the set of features that yielded best predictive models.

All calculations (texture computation and statistics) were done with Matlab (Matlab R2011b, MathWorks, Natick, MA, USA). Open source codes “KeyRes-Technologies” and “grayrlmatrix” under Matlab were used for computing texture parameters. The software Image J (http://rsbweb.nih.gov/ij/) was used for the segmentation of the ROIs. A *p*-value < 0.05 was considered as statistically significant for all tests cited above, as the universal null hypothesis was of no interest in this study [45].

## Results

### Biological and imaging parameters

Morphological, biological and histological findings are reported in Table 2. Neither the mass enhancement nor the non-mass enhancement were statistically different between NR and PR + CR. NR were significantly more represented in Luminal-A subtype compared to PR + CR. NR were significantly less represented in Ki67 > 14 % and HR-/HER2+ compared to PR + CR (non-significant trend). No statistical difference on histological grade between NR and PR + CR was observed.

Texture and kinetic parameters are reported in Table 4. Significant differences between healthy tissues and malignant tissues were observed for all texture parameters (all *p*-value < 0.05).

**Table 4:** Median values (95 % CI) of the texture and kinetic parameters

	Normal tissue	CR + PR	NR	*p-*value^a^
Energy	58 [44; 74]	36 [33; 41]	45 [42; 55]	7.9 10^−5^
Entropy	157 [141; 172]	187 [181; 193]	175 [165; 180]	6.4 10^−5^
Contrast	8 [6; 10]	14 [11; 16]	13 [10; 16]	8.6 10^−5^
Homogeneity	165 [150; 176]	140 [134; 146]	149 [144; 156]	5.1 10^−5^
Correlation	22 [18; 29]	47 [42; 52]	47 [44; 50]	1.8 10^−14^
Inv. Diff. Moment	174 [161; 185]	148 [141; 153]	158 [153; 165]	4.2 10^−5^
Sum average	69 [65; 76]	119 [114; 124]	120 [109; 127]	3.6 10^−19^
Sum variance	70 [60; 76]	92 [88; 99]	97 [86; 110]	2.2 10^−15^
Difference variance	74 [67; 81]	87 [82; 93]	80 [78; 83]	4.6 10^−3^
SRE	0.009 [0.008; 0.009]	0.004 [0.0039; 0.0044]	0.0038 [0.0035; 0.0047]	6.2 10^−19^
LRE	126 [114; 144]	266 [246; 279]	284 [229; 309]	7.6 10^−20^
GLN	158 [137; 229]	432 [338; 589]	416 [298; 817]	1.2 10^−10^
RLN	71 [58; 86]	111 [74; 120]	105 [89; 205]	6.2 10^−4^
RP	0.68 [0.62; 0.72]	0.72 [0.71; 0.75]	0.70 [0.66; 0.73]	9.3 10^−4^
LGRE	0.75 [0.71; 0.78]	0.79 [0.78; 0.81]	0.77 [0.74; 0.80]	9.8 10^−4^
HGRE	3.11 [2.54; 3.99]	2.55 [2.33; 2.76]	2.83 [2.49; 3.34]	8.8 10^−3^
SRLGE	0.0060 [0.0056; 0.0067]	0.0033 [0.0031; 0.0034]	0.0030 [0.0028; 0.0036]	2.6 10^−17^
SRHGE	0.028 [0.024; 0.034]	0.011 [0.010; 0.012]	0.011 [0.009; 0.014]	6.4 10^−18^
LRLGE	93 [84; 101]	204 [189; 215]	214 [177; 251]	6.0 10^−20^
LRHGE	412 [343; 509]	679 [615; 745]	799 [592; 925]	1.9 10^−9^
Amplitude	_	75 [70; 80]	68 [59; 79]	_
Wash-out	_	0.04 [0.03; 0.06]	0.04 [0.008; 0.070]	_
Wash-in	_	0.72 [0.64; 0.83]	0.63 [0.42; 0.70]	_

Amplitude is given in arbitrary unit (AU), wash-in and wash-out in AU.s^−1^

*NR* Non response, *CR* Complete response, *PR* Partial response

^a^Statistical difference (Wilcoxon) between normal tissues and tumoral lesion

### Mono-parametric prediction

AUC values, sensitivity and specificity of selected cut-offs are given for all parameters in Table 5. Parameters energy, entropy, homogeneity inverse difference moment, RP, HGRE and wash-in were found to have an AUC significantly different from 0.5 (*p*^energy^ = 0.002, *p*^entropy^ = 0.003, *p*^homogeneity^ = 0.001, *p*^inv. diff. mom.^ = 0.001, *p*^diff. var.^ = 0.023, *p*^RP^ = 0.045, *p*^HGRE^ = 0.038, *p*^wash-in^ = 0.008). The performance associated with these parameters ranged from fair (0.5 < AUC ≤ 0.7) to good (0.7 < AUC ≤ 0.9). The pairwise comparison of AUCs did not allow ranking strictly these parameters according to their individual performance (*p* > 0.05 whatever the comparison).

**Table 5:** Performance of the individual parameters measured from ROC curves (based on the Youden index for determining cut-offs)

	Se (%)	Sp (%)	AUC	Cut-offs
Energy^a^	64	79	0.702	41
Entropy^a^	64	79	0.696	182
Contrast	30	95	0.576	17
Homogeneity^a^	58	84	0.701	144
Correlation	62	16	0.512	42
Inv. Diff. Moment^a^	60	84	0.711	152
Sum average	28	90	0.527	103
Sum variance	78	42	0.583	104
Difference variance^a^	60	79	0.649	86
SRE	80	42	0.569	0.004
LRE	86	37	0.569	301
GLN	74	42	0.555	621
RLN	38	90	0.579	75
RP^a^	42	90	0.640	0.740
LGRE	42	90	0.630	0.800
HGRE^a^	42	90	0.644	2.40
SRLGE	70	53	0.582	0.003
SRHGE	16	100	0.510	0.007
LRLGE	80	37	0.536	233
LRHGE	72	58	0.620	781
Amplitude	67	58	0.567	69.1
Wash-out	27	95	0.594	0.09
Wash-in^a^	86	47	0.685	0.50
Mass^b^	63	46	0.546	_
non Mass^b^	63	46	0.546	_
Ki67 > 14 %^b^	42	82	0.621	_
HER2 +^b^	79	44	0.615	_
HR-/HER2 +^b^	100	20	0.600	_

An overall better performance of GLCM compared to RLM parameters, as well as a better performance of texture and kinetic parameters compared to BI-RADS and biological parameters was observed

^a^Parameters performing significantly better than a random classifier (p^(AUC > 0.5)^ < 0.05)

^b^Categorial variables without cut-offs

### Multi-parametric prediction

Using *k*-means clustering as classifier, a predictive model relying on four parameters (inverse difference moment, GLN, LRHGE, wash-in) was found to perform with a predictive accuracy of 68 %: Se = 84 % (16/19 NR) and Sp = 62 % (31/50 PR + CR). Using log-transformed parameters (energy, homogeneity, wash-in, LRHGE), it was possible to increase the percentage of NR classified correctly up to 95 % (18/19), but with a lower specificity of 32 % (16/50 PR + CR) and a lower predictive accuracy of 64 %. Using logistic regression as classifier, a more parsimonious predictive model was found. It was based on two texture parameters only (homogeneity, LGRE) and exhibited a predictive accuracy of 74 %: Se = 74 % (14/19 NR) and Sp = 74 % (37/50 PR + CR). Models using other combinations and/or a larger number of parameters did not improve the predictive accuracy (regardless of the type of classifier).

## Discussion

The first observation of this study is that texture analysis discriminates healthy breast tissues from tumor lesion. Texture is more heterogeneous and coarse in the enhancing part of the lesion compared to healthy tissue. This observation agrees with previously published results on the ability of visual texture parameters to differentiate normal from malignant tissue with breast DCE-MRI [27].

The second observation is that the predictive performance of individual texture and kinetic parameters did not exceed the level fair, except for parameters homogeneity and inverse difference moment whose performance level is evaluated as good.

The third observation is that a multi-parametric model based on texture and kinetic parameters was able to predict non-response to NAC with a good performance level. This observation needs to be discussed according to the study design.

The usefulness of pre-NAC DCE parameters in predicting response to NAC was proven in several studies, however on the basis of different assumptions. While Uematsu *et al.* [24] suggest that a washout enhancement pattern is related to a more effective cycle of NAC, Pickles *et al.* [25] conclude that high values of perfusion and capillary permeability indicate a high level of angiogenesis and, are therefore indicative of treatment failure. In our study, a faster contrast agent uptake by the tumor as well as a (non-significant) trend towards a higher washout value were observed in PR + CR. The increased pre-NAC vascularity and permeability characteristics may be interpretable in terms of better delivery of chemotherapeutic agents to the tumor and better treatment efficacy. However, we think that the assumption of vascular characteristics associated with NAC efficacy must be considered with caution. First, drug resistance is a multifactorial phenomenon where cellular mechanisms have a predominant role [46]. Secondly, standard protocol in dynamic breast MRI based on a high spatial resolution such as the one we used in this study does not meet all requirements for an accurate analysis of transport mechanisms in lesions [47]. Such analysis requires a sampling rate less than the mean transit time of the contrast agent, the measurement of an individual arterial input function, the knowledge of the relationship between signal intensity and contrast agent concentration in the tissues and a pertinent mass transport model [48–50].

The usefulness of pre-NAC texture parameters in predicting response to NAC was confirmed in this study, but based on a partially different set of parameters compared to previously published studies. In [33], an increased heterogeneity of the texture indicated by the higher values of two parameters (contrast, difference variance) was observed in NR. However, texture was evaluated from the whole lesion including central necrosis, thus increasing the heterogeneity measurements. In the present study, a reduced heterogeneity of the texture (as indicated by the four significant GLCM parameters) in the enhancing part of the lesion was found in NR compared to PR + CR. One of these parameters (inverse difference moment) was found to be predictive of a reduced chemotherapeutic response, but jointly with two RLM parameters (GLN, LRHGE) whose high values indicate a more heterogeneous distribution of some grey level run lengths in NR. There is no obvious explanation at the histological level for these differences of behavior. Further investigations on how and which texture parameters are associated with tumor biology may help defining on the relationship between texture heterogeneity and response to NAC.

Methodological differences in the assessment of texture limit the comparisons between studies. The most common texture analysis techniques are derived either from grey level histogram [51], gradient matrix [52], GLCM [29], RLM [40], local binary patterns [52], auto-regressive model [53], Riesz transform [54], multiple frequency scales [55], S-transform [56] or from wavelet [57]. None of these approaches is superior to the others since their effectiveness basically relies on the visual properties of images to which they are applied. Combining various texture methods may improve the characterization of breast lesions as demonstrated by our data. However, increasing the number of texture parameters has some drawbacks. Dimensionality reduction techniques prior to classification, sophisticated machine learning classifiers as well as larger training datasets become necessary. Our four-parameter predictive model may thus present a practical advantage over those proposed in [33, 34] for further testing.

The usefulness of BI-RADS mass/non-mass enhancement could not be validated possibly due to a high prevalence of non-mass lesions in our cohort [8, 24]. Rates of complete responders are known to be different within tumor subtypes [7]. We assumed that the low statistical power induced by the small number of patients within each subtype prevents from observing such difference. Ki67 > 14 % and HR-/HER2+ were the only markers more often seen in responders. These parameters, having a fair performance, were not retrieved in the best predictive model.

The performance of our predictive model, albeit good, appeared lower compared to the one reported in previous studies. In [26, 32, 34], predictive accuracy was 85, 83 and 88 % respectively. However, comparison is flawed as cross-validation was not performed in either of these studies, while it is necessary to get an unbiased estimate of the predictive accuracy [58]. The use of techniques such as cross-validation, bootstrapping or Bayesian confidence interval should be generalized to get a reliable assessment of classifier performance, useful to estimate the relevance of the working hypothesis and mandatory for clinical acceptance.

Clinical response definition and chemotherapy regimen may influence the predictive accuracy. In [32], the difference between ‘good’ and ‘bad’ responders was arbitrarily fixed at 50 % decrease in tumor volume between baseline MRI and after 2 cycles of chemotherapy. We on the other hand used the pathological response, which is the gold standard in the assessment of response to NAC. In [34], the predictive accuracy of the model depended on the type of chemotherapy regimen undergone by the patients. A similar report was made by Richard *et al.* studying the predictive value of pre-treatment apparent diffusion coefficients [59]. This raises the question of whether a generalized predictive model of response to NAC independent of chemotherapy regimen can be established.

There are several limitations to the study. First, this is a retrospective study based on a limited number of patients. While our first dataset served for model learning, a second and larger dataset is necessary to validate the performance of the predictive model. This approach, replicating the most interesting results of the pilot study, will address significance problem that may arise when dealing with a large set of parameters. Besides, various types of machine learning classifier can be envisaged, influencing the performance as well [60]. Further tests may be needed to compare the efficacy and practicality of these classifiers. In this pilot study, a single subtracted MR image was evaluated at a specific time-point corresponding to the enhancement peak on intensity time curves. Subtracted images were chosen because of the attenuation of the normal parenchymal background enhancement. Tests on late time points (i.e. on the fifth and sixth dynamics corresponding to imaging of tumor permeability) did not allow for the identification of a good predictive model. Due to its complexity, multi-slice evaluation based on 3D segmentation of the lesion and 3D texture analysis was not envisaged in first instance. However, 3D is one of the strategies to be considered for improving the prediction of response to NAC. Only patients with invasive ductal carcinoma were included. The choice of a single subtype of cancer, far from constituting a selection bias, is legitimate within a dichotomous approach of the problem of predicting response to NAC. Our outcome score depended on histopathological findings and we wanted therefore to obtain a histologically homogeneous group to test texture analysis. Furthermore, it has been demonstrated that invasive lobular carcinoma is less sensitive to NAC [61]. Other studies emphasized that in ILC, immediate treatment with endocrine therapy might be more beneficial [62]. Finally, though combining texture and kinetic parameters with BI-RADS and biological markers did not presently improve the predictive accuracy, these latter parameters should not be discarded in another framework where different (or several) subtypes of breast cancer would be studied.

## Conclusion

Pre-NAC texture and kinetic parameters measured from dynamic breast MRI help predict non-response of invasive ductal carcinoma to neoadjuvant chemotherapy. Due to the numerous steps necessary to the processing of DCE-MR images, further investigations are needed. It is especially important to test other texture features and statistical classifiers to improve the overall performance of the model, and to include larger groups of tumor subtypes in order to improve the generalization properties of the predictive model. The rationale behind these investigations is the development of a computer-assisted prediction solution dedicated to breast MRI. Such a solution would be cost-effective in comparison to genetic/molecular assessments and may contribute to an appropriate treatment outcome for patients with breast cancer initially eligible for NAC.
